# Acquired Radial Club Hand: An Algorithm to Manage Radial Deficiency

**DOI:** 10.7759/cureus.43154

**Published:** 2023-08-08

**Authors:** Husnain Khan, Nur Ul Ain, Kamal Afridi, Zahid Bhatti, Umer Chishti, Tayyab S Malik, Tahseen Cheema

**Affiliations:** 1 Plastic and Reconstructive Surgery, Holy Family Hospital, Rawalpindi, PAK; 2 Plastic Surgery, Bolan Medical College, Quetta, PAK; 3 Plastic Surgery, Wahid Trust Hospital, Gujrat, PAK; 4 Orthopedic Surgery, National Orthopedic & General Hospital, Bahawalpur, PAK

**Keywords:** malunion, trauma, osteomyelitis treatment, orthopaedic hand surgery, upper limb, radius, radial club hand, osteomyelitis of radius, radial deficiency, acquired radial club hand

## Abstract

Introduction

Loss of radius either due to trauma or infection results in a deformity resembling a congenital radial club hand. This deformity results in difficulty to perform hand functions and cosmetic appearance and is called acquired radial club hand. There are a few case reports for the treatment of this severe deformity, but there are no proper guidelines for the management of this disease. From our experience, we decided to provide treatment guidelines for acquired radial club hand.

Objectives

To evaluate the outcome of radial deformity treatment in acquired radial club hand injuries and develop a treatment algorithm.

Patients and methods

It is a case series study of 11 patients with acquired radial club hand. It was conducted at a tertiary care hospital in Pakistan, from year 2016 to 2022. Basic principles of management of infection and trauma were followed. For the treatment of radial deformity, different options were opted according to the type of deformity, following the principles of treatment of congenital radial club hand. The outcome was graded on functional activity, pain, and bony union.

Results

Out of 11 patients, 36.36% showed excellent results, 27.27% showed good results, 27.27% showed fair results, and 9.09% showed poor results. Results were excellent in all patients with avascularized bone graft and distraction lengthening, with or without the Darrach procedure. Of the patients in whom distraction lengthening was performed, one patient showed excellent results while the other patient achieved similar results after the Darrach procedure of ulnar shortening. In the case of one bone formation by radioulnar synostosis, the results were variable. Two of the patients showed good outcomes while the other two had fair outcomes. Results in the case of ulnar centralization were mixed with good, fair, and poor results in one patient each. After three months of follow-up, 87% of the patients showed fair to excellent results.

Conclusion

With our experience, we recommend an algorithm for the treatment of acquired radial club hand.

## Introduction

Loss of radius either due to trauma or infection results in volar subluxation and radial deviation of the wrist. This deformity resembles a congenital radial club hand and is called an acquired radial club hand. It is a complex problem because there can be malunion of the distal radial fractures, issues of bone loss, persistent infection, lack of functional hand and wrist mobility, limb length inequality, and appearance that need to be addressed. Basic principles of treatment of infection and trauma should be followed before planning for the final reconstructive plan. Once the wound is healthy, various treatment options have been mentioned to correct and overcome the radial deficiency. These include cancellous [1,2] or structural non-vascularized bone grafting, open reduction internal fixation of the radius with ulnar shortening [3,4], creation of a one-bone forearm [5-8], use of vascularized fibula, bone transport using an external fixator, and centralization of the ulna. Due to the rarity of the disease, there are no proper guidelines to manage this difficult problem. Different surgeons have opted for different options on different occasions. There is no single study, as far as our knowledge, that can provide a treatment protocol for the correction of a radial deficiency in acquired radial club hand. So, from our previous experience, we decided to provide an algorithm to manage the radial deficiency.

## Materials and methods

**Figure 1:**
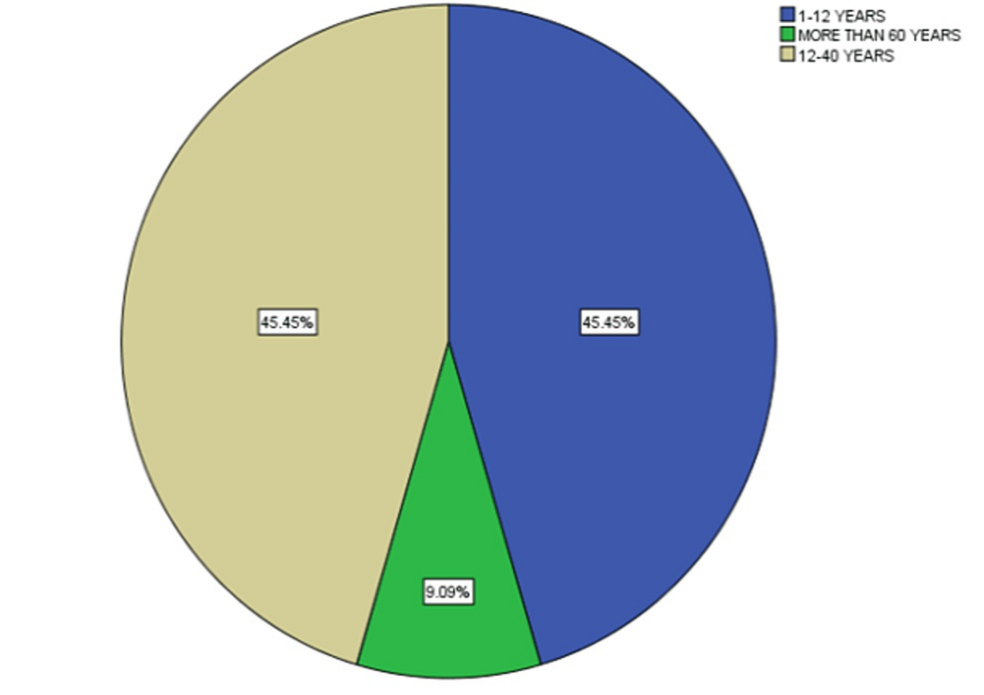
Age distribution of participants

The study was conducted at a tertiary care hospital in Pakistan over a period of seven years. It was a case series study in which a total of 11 patients (six females and five males) were included. Patients were divided into three groups according to age: Group A (age range of one to 12 years), Group B (from 12 years to 60 years), and Group C (more than 60 years). Out of these, 45.5% were children, 45.5% were between the age of 12-60 years, and 9% were more than 60 years old (Figure 1).

Inclusion criteria were mainly individuals who have been clinically diagnosed with acquired radial club hand, a condition characterized by the partial or complete absence of the radius in the forearm. Whereas, the exclusion criteria consisted of congenital radial club hand, other significant upper limb deformities, and cognitive or communication impairments.

**Figure 2:**
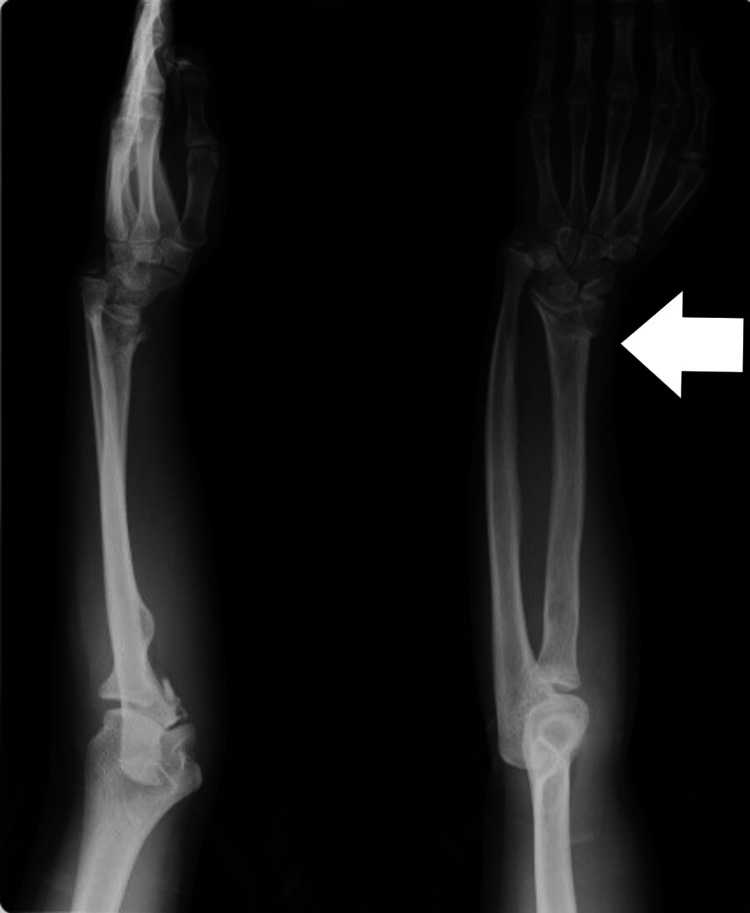
Short radius with no segmental loss

**Figure 3:**
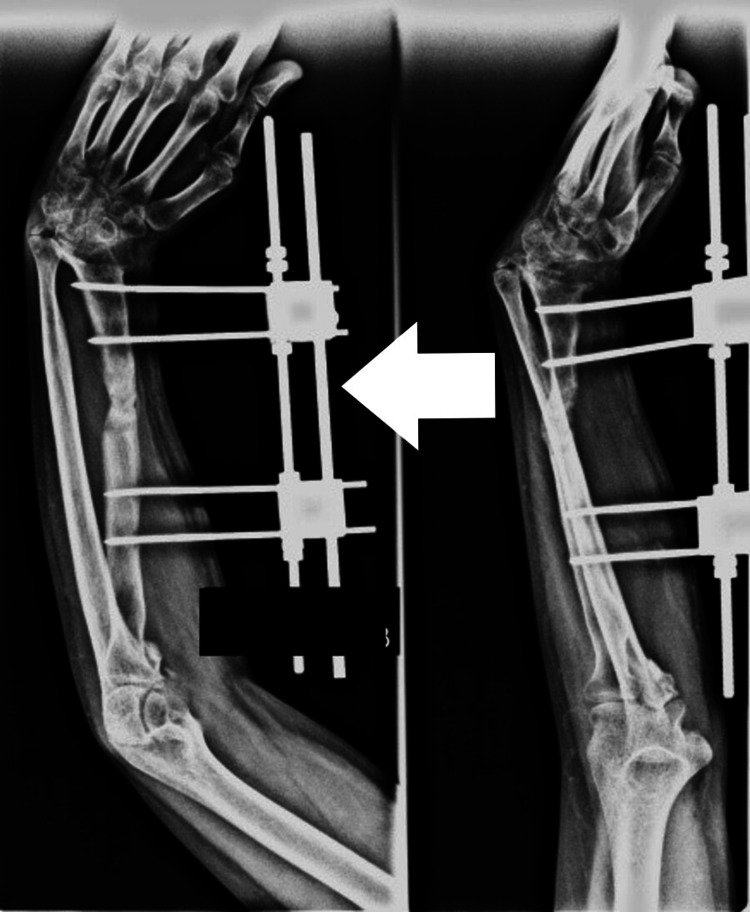
Distraction lengthening of radius

**Figure 4:**
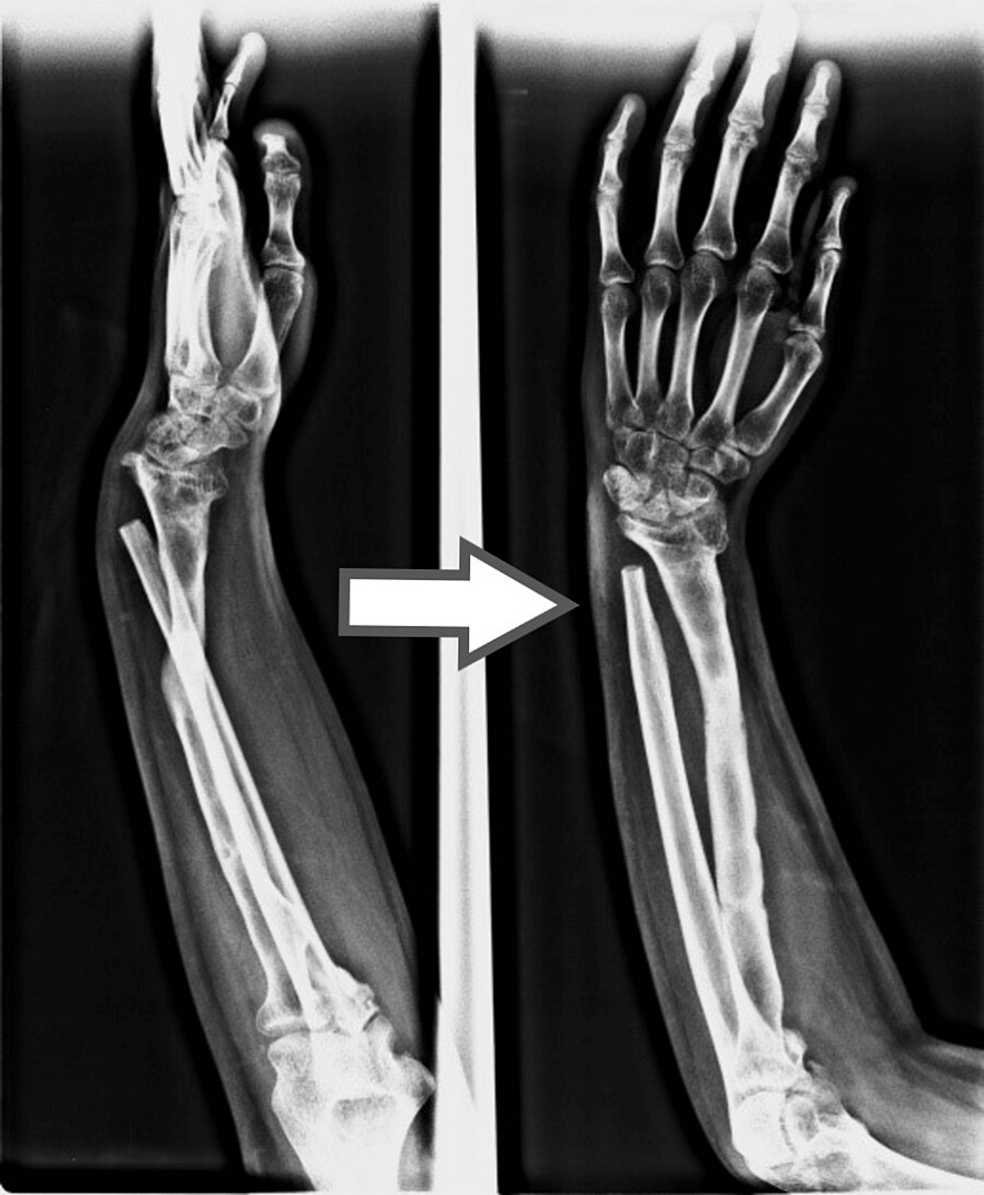
Darrach procedure of ulnar shortening

There were two patients in whom radius was short but there was no segmental loss. Both these patients presented late after the management of radial fractures due to a road traffic accident (RTA). Radial shortening was possibly due to growth arrest of the radius or infection. Distraction lengthening was performed in both of these patients by a uniplanar distractor. Single-segment distraction was done by creating an osteotomy and applying pins on the proximal segment, at a rate of 1 mm/day in three divided sessions, i.e., 0.33 mm every eight hours. It was initiated after waiting for a latency period of one week. Distraction was continued until the desired length was achieved. Once the normal length of the forearm was achieved and radial deviation and volar subluxation were corrected, distraction was stopped, and the external fixator was left in place until consolidation occurred, which was confirmed on X-ray (Figures 2-4).

In one patient, the ulna was still long after distraction causing difficulty in wrist movement. Darrach procedure of ulnar shortening was performed in this patient (Figure 4).

**Figure 5:**
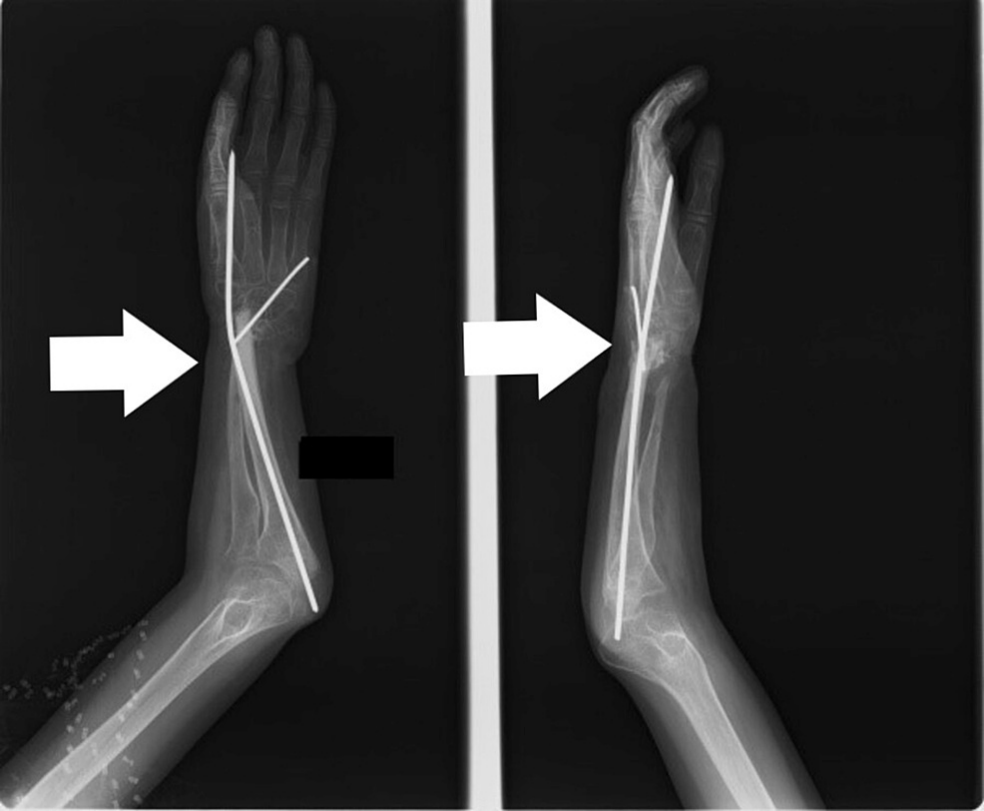
Centralization of ulna

Three patients presented with the scenario of a total loss of a segment of the distal radius. All these patients had a history of motorbike accidents. After debridement, there was a loss of distal radius. In one patient, a para-umbilical perforator flap (abdominal flap) was done to cover the defect before bony correction. Once the infection settled and soft tissue coverage was provided, there were two treatment options, i.e., either to reconstruct the radius with a free vascularized fibular head graft or centralization of the ulna. Considering the economic condition of the patients, centralization of the ulna was done in all three patients with K-wire fixation (Figure 5).

**Figure 6:**
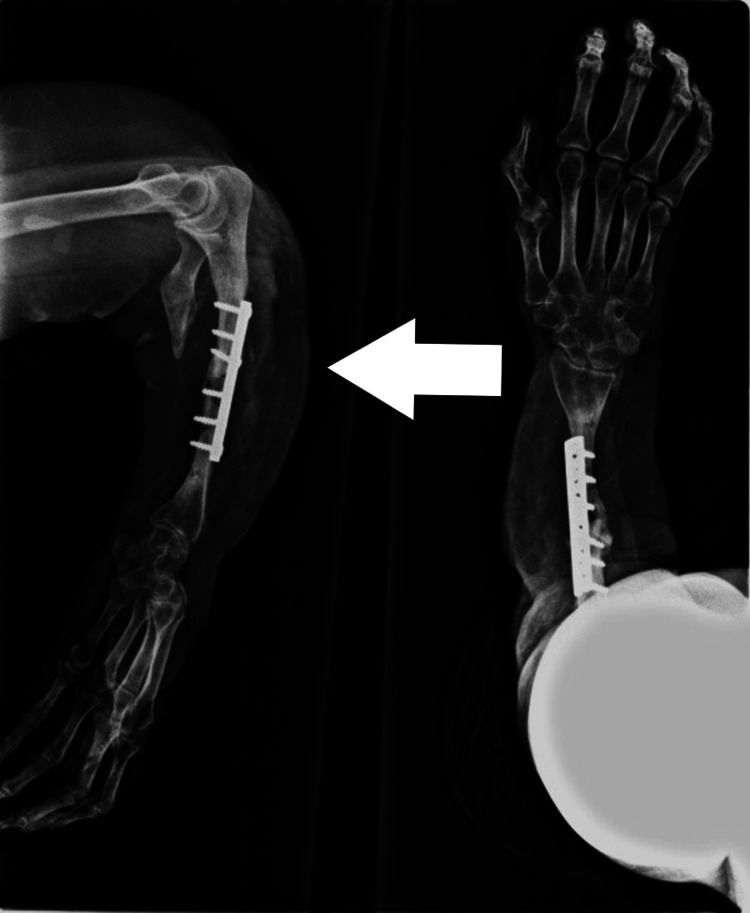
Radioulnar synostosis

Four patients presented with segmental loss of radius in which distal radius or a remnant of distal radius was present while the proximal segment was missing. Three patients had a history of a motorbike accident and one patient had a history of a fall. The patient with a history of a fall was first managed by an orthopedic colleague with plating of the radius, which resulted in infection and later osteomyelitis of the shaft of the radius. Three patients with a history of RTA were first managed in the accident and emergency department and then referred to us. In stage 1, only debridement was done. Once the wound was healthy, all patients were assessed for soft tissue deficiency. In one patient, we performed a groin flap to cover the defect before correcting radial deficiency while in the other three patients, we were able to close the skin primarily, so radial deficiency was corrected in the same setting. In two patients, most of the proximal radius was missing or there was a small remnant of radius, so we performed radioulnar synostosis by plating the distal radius and proximal ulna (Figure 6).

**Figure 7:**
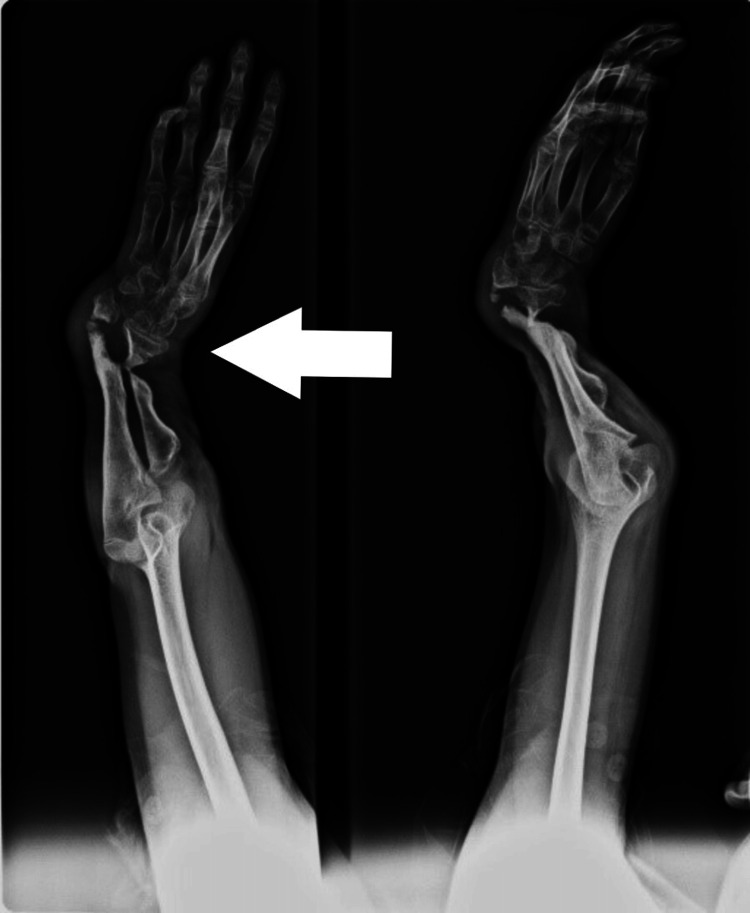
Only a fragment of distal radius present

**Figure 8:**
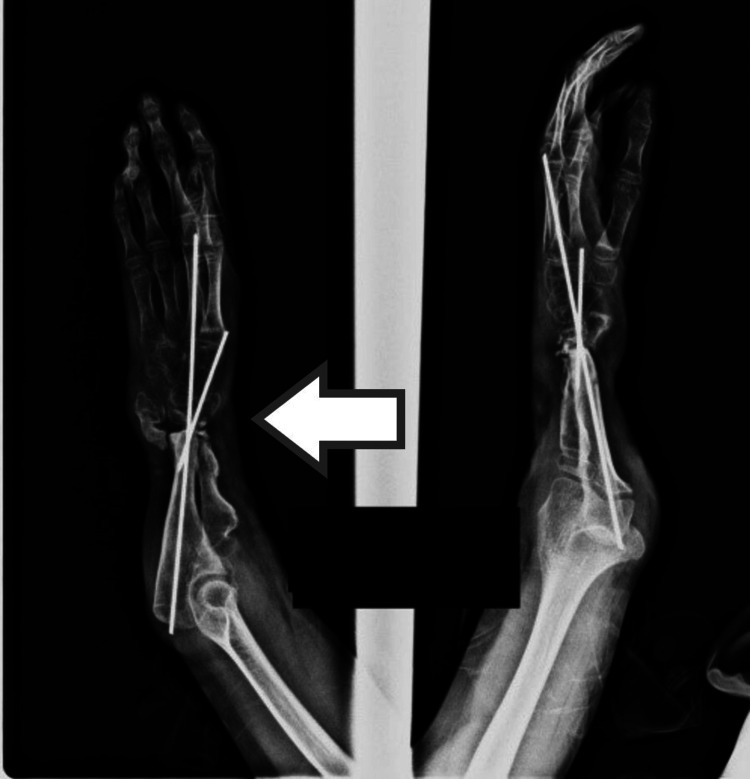
Fusion of ulna with distal radius by wires

In two children, the plating was not possible due to a small distal fragment of radius, so we fixed the proximal end of the ulna to the distal radial fragment by K-wire in a side-to-side fashion (Figures 7, 8). The epiphyseal plate was preserved, therefore, growth will continue. In case of any shortening in the future, nothing needs to be done unless there is a functional deficit, in which case distraction osteogenesis can be done to achieve the desired length. Pronation and supination movements were lost as a single bone is present instead of two bones. Moreover, for any further deficit, tendon transfer or other secondary procedures can be done eventually.

**Figure 9:**
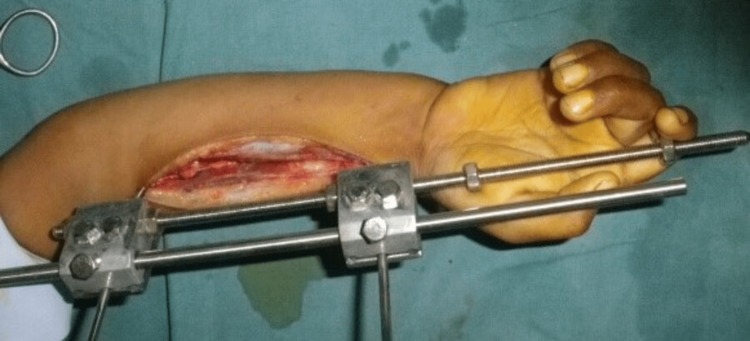
Distraction lengthening

**Figure 10:**
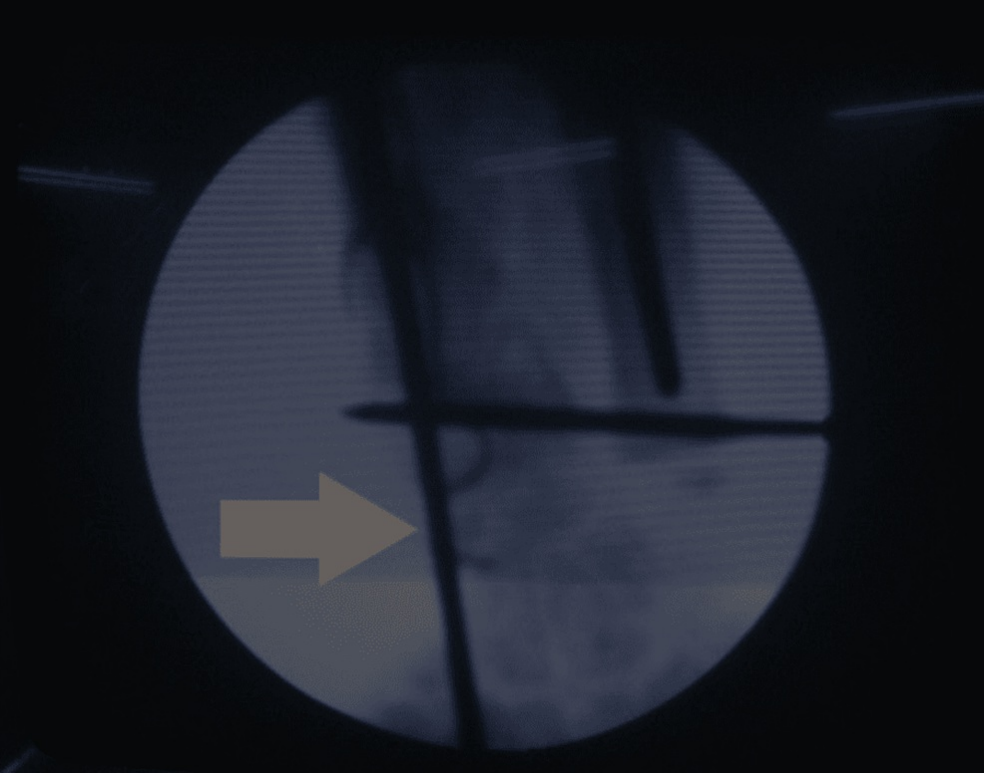
Assessment of normal wrist joint space after distraction lengthening (on fluoroscopy)

**Figure 11:**
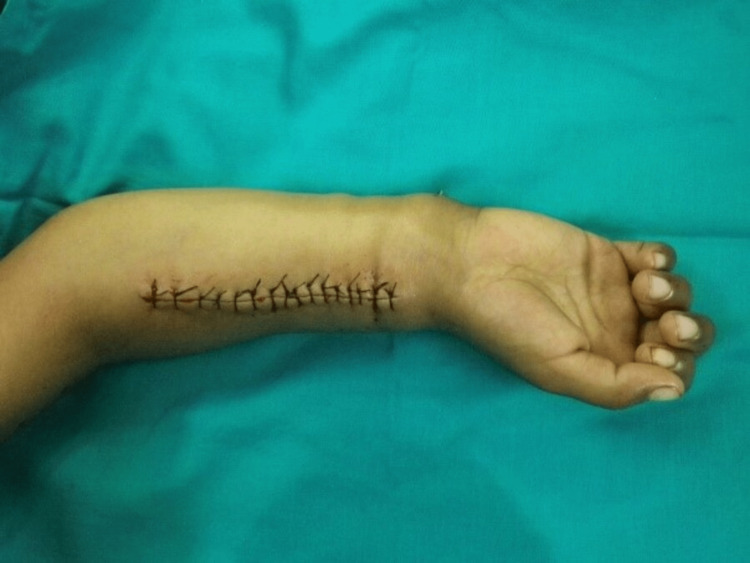
Primary wound closure after avascularized bone graft

Two patients presented with segmental loss of the radial shaft. It was possible to preserve the distal radioulnar joint. There was a loss of about 4 cm of radius in one patient and 6 cm in the other patient. After debridement, the distraction of the radius was done to achieve the normal relation of the radius and ulna at the wrist level. Once the normal anatomy was achieved, an avascularized bone graft from the fibula was used to fix the intervening radial deficiency (Figures 9-11).

Evaluation of results

The first follow-up was at two weeks for stitch removal, the second follow-up was at four weeks for backslab removal, and after ensuring bony continuity and callus formation, active mobilization and physiotherapy were initiated. Clinical outcome at three-month follow-up after the final procedure was measured and compared using a 10-point, subjective scoring system designed for this review, based on function, pain, and attainment of union (Table 1).

**Table 1:** Ten-point scoring system

Variable	Level	Points
Functional activity	Near normal	6
	Complex activities	4
	Activities of daily life	2
	Minimal activities	0
Pain (visual analog scale)	No pain (0)	3
	Mild pain (1-3)	2
	Moderate pain (4-6)	1
	Severe pain (7-10)	0
Bony union	Yes	1
	No	0

The functional capability was assessed as follows: "near normal" patients reported no significant impairment in their use of the involved forearm. "Complex activity" function included those patients who reported definite impairment in forearm performance but who could still easily perform tasks beyond the "daily activity" level (e.g., manual labor, repetitive motor tasks, and lifting more than 20 lbs. repetitively). The "activities of daily living" function allows patients to perform basic personal hygiene, drive a car, cook, and so on. The "minimal" function describes the forearm as useless for most or all activities of daily living.

Pain level was subjectively graded by patients according to a 10-point visual analog scale. The pain was graded into none (VAS = 0), mild (VAS = 1-3), moderate (VAS = 4-6), or severe (VAS = 7-10). Union was judged by single-reviewer analysis of serial anteroposterior and lateral X-ray films. Overall outcomes were then graded by total score summation into excellent, good, fair, and poor (Table 2).

**Table 2:** Final outcome

Cumulative score	Grading
8-10	Excellent
6-7	Good
4-5	Fair
0-3	Poor

## Results

Table 3 shows the demographic details such as age, gender, type, and outcome of radial deficiency of the patients included in this study.

**Table 3:** Demographic and outcome of radial deficiency RTA: road traffic accident.

Cases	Age group	Sex	Affected side	Cause of radial deficiency	Radial deficiency	Procedure performed
1	Child	F	Right	History of osteomyelitis after bony fixation	Loss of distal segment of the radius with radioulnar joint not preservable	One bone formation with distal radioulnar synostosis
2	Old	F	Right	RTA, motorbike accident	Loss of complete distal radial segment	Centralization of ulna
3	Young	M	Right	RTA, motorbike accident	Loss of segment of the radial shaft, distal radioulnar joint preservable	Avascularized bone graft
4	Young	M	Right	RTA	Short radius without segmental loss	Distraction lengthening with the Darrach procedure
5	Child	M	Right	RTA, motorbike accident	Loss of distal segment of the radius with radioulnar joint not preservable	One bone formation with distal radioulnar synostosis
6	Child	M	Right	RTA, motorbike accident	Loss of complete distal radial segment	Centralization of ulna
7	Young	F	Right	RTA	Loss of distal segment of the radius with radioulnar joint not preservable	One bone formation with distal radioulnar synostosis
8	Young	F	Left	RTA	Short radius without segmental loss	Distraction lengthening
9	Child	F	Left	Fall from height	Loss of complete distal radial segment	Centralization of ulna
10	Child	M	Right	RTA	Loss of distal segment of the radius with radioulnar joint not preservable	One bone formation with distal radioulnar synostosis
11	Child	F	Right	RTA	Loss of segment of the radial shaft, distal radioulnar joint preservable	Avascularized bone graft

Out of 11 patients, 36.36% showed excellent results, 27.27% showed good results, 27.27% showed fair results, and 9.09% showed poor results. Results were excellent in all patients with avascularized bone graft and distraction lengthening, with or without the Darrach procedure. Both patients with an avascularized bone graft can perform the routine activities of life with near normal competence and without much pain, and follow-up X-rays showed a good bony union of the fibular graft with proximal and distal ends of the ulna. The reason for an excellent outcome in these cases was that only a small portion of the radius was missing, which was replaced and a normal relationship of the ulna and radius was maintained at both elbow and wrist. In patients in whom distraction lengthening was performed, one patient showed excellent results while the other patient achieved similar results after the Darrach procedure of ulnar shortening. In the case of one bone formation by radioulnar synostosis, the results were variable. Two of the patients showed good outcomes as they can perform complex activities of life like driving, holding weight without significant pain, and good bony union. Similarly, the other two patients can perform the activities of daily life, such as holding objects, dressing and undressing, eating with hands without major pain, and adequate bony union, fitting into the fair outcome category. Results in the case of ulnar centralization were mixed with good, fair, and poor results in one patient each (Table 4).

**Table 4:** Outcome of the procedure using a 10-point scoring system

Case	Procedure	Function	Pain	Bony union	Score
1	Distal radioulnar synostosis	2	2	1	Fair
2	Ulnar centralization	2	2	1	Fair
3	Avascularized bone graft	6	3	1	Excellent
4	Distraction lengthening + Darrach procedure	6	3	1	Excellent
5	Distal radioulnar synostosis	2	3	1	Good
6	Ulnar centralization	2	3	1	Good
7	Distal radioulnar synostosis	2	3	1	Good
8	Distraction lengthening	4	3	1	Excellent
9	Ulnar centralization	0	0	1	Poor
10	Distal radioulnar synostosis	2	3	1	Fair
11	Avascularized bone graft	4	3	1	Excellent

## Discussion

Although acquired radial club hand is not a common disease yet, it is not an unknown terminology. Dreyfuss [9] reported a case report of an acquired club hand due to osteomyelitis and identified a deformity similar to a congenital radial club hand. A lot of case reports on acquired radial club hands have been published. As it is a rare deformity, so different treatment options have been mentioned in the literature.

Honsy [10] mentioned the distraction of the radius with the Ilizarov technique in three patients with a non-union of radius. In two patients, he found it to be a safe technique to correct traumatic radial club hand. In 2007, Zhang and colleagues [11] found good cosmetic and functional results in 12 patients with acquired radial club hand after osteomyelitis. Their method of distraction lengthening was with a monolateral fixator.

Ulnar centralization for the treatment of this deformity has also been mentioned by different surgeons with different techniques. Malki and colleagues [12] performed centralization of the ulna by the modified Hay Groves procedure in 2000. They presented a case report on the treatment of an 11-year-old boy with an infected non-union of the radius with extensive bony loss. Kanojia and colleagues [13] first performed distraction of the ulna with an external fixator and then centralization of the ulna in an infantile patient with acquired radial club hand along with septic dislocation of the elbow joint.

At the same time, some of the surgeons recommended one bone formation as a treatment option for acquired radial club hand. Rasool [14] performed one bone formation by radioulnar synostosis in six children by using intramedullary pins. However, if the bony gap between the proximal ulna and distal radius is extensive, one bone formation can also be accomplished by using vascularized or non-vascularized bone graft as described by Peterson et al. [15] and Arai et al. [16].

Vascularized and non-vascularized bone grafts to fill the radial deficiency by keeping the ulna intact have also been mentioned as a treatment modality. Netrawichien [3] described good functional and cosmetic results at follow-up at one year in two patients with acquired club hand deformity, which had been treated by cancellous bone graft and plating combined with ulnar shortening. Dugdale et al. [1] described cortico-cancellous bone grafting of the radial defect after complicating acute hematogenous osteomyelitis in an infant. The vascularized fibular graft was used by Jupiter et al. [17] for segmental radial deficiency. Some authors mentioned combined treatment modalities for this deformity, such as Sabharwal [18], who described bone transport with Ilizarov and filling the gap with a bone graft.

**Figure 12:**
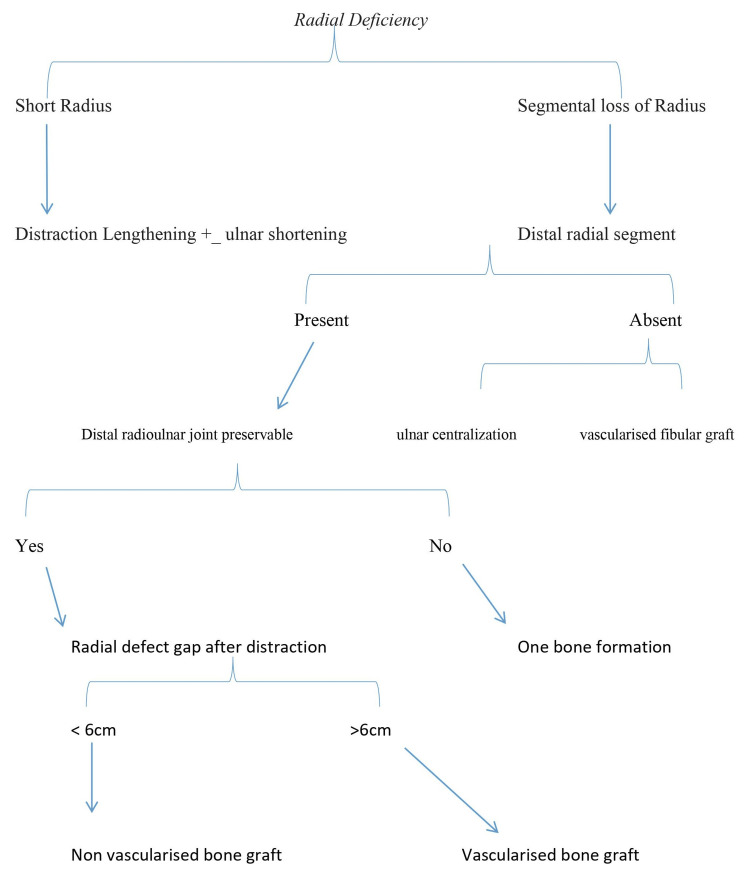
Algorithm for treatment of radial deficiency in acquired radial club hand

As the disease is uncommon and radial deficiency is variable, so there are no clear guidelines to manage this difficult problem. From our experience, we have tried to sort out this problem. We found that radial deficiency is a broad term, so we divided it into two parts, that is, either there is a short radius or the segment of radius is absent. For short radius, distraction lengthening of the radius with or without ulnar shortening should be performed. Treatment of segmental loss of radius is further dependent on whether the distal part of the radius is present or absent. If the distal part of the radius is absent, then one should either go for centralization of the ulna or a vascularized fibular graft. We did not perform the latter option in any of our cases due to the poor economic conditions of our patients but a free vascularized fibula is an option that should be kept in mind for the reconstruction of the distal part of the radius. In segmental loss of radius, if the distal part of the radius is present, then the management depends upon whether the distal radioulnar joint is preservable or not. If the joint is not preservable, then one bone formation with radioulnar synostosis is a better treatment option. If it is possible to preserve the joint, then management depends upon whether the defect is up to 6 cm or more. If the defect is up to 6 cm, a non-vascularized graft either cortical or cortico-cancellous can be used to fill the radial gap. If the radial defect is more than 6 cm, then a free vascularized fibular graft should be used as a treatment modality. Although we have not performed free vascularized fibular graft in any of the patients yet, we recommend the following algorithm should be followed to treat radial deficiency in acquired radial club hand (Figure 12).

## Conclusions

Acquired radial club hand is a distinct entity from congenital radial club hand. Acquired radial club hand can be secondary to trauma, infection, tumor, etc. Due to the presence of these causes, the management plan, treatment options, and guidelines also vary from that of congenital radial club hand. First priority is to achieve soft and supple tissue coverage to achieve tissue equilibrium. Then, the defect is treated as required. There was a lack of any algorithm to manage such cases; hence, we proposed one based on our experience of managing such cases. This will help in more efficient and effective management of cases of acquired radial club hand.
